# Repair of a finger pulp or fingertip defect using a palmar rotatory flap pedicled with the perforating branch of the proper palmar digital artery: a retrospective study

**DOI:** 10.1186/s13018-023-04156-y

**Published:** 2023-09-13

**Authors:** Yongjun Du, Zhongfeng Cui, Shaoquan Pu, Zhi Peng, Sheng Lu

**Affiliations:** 1 Kunming Medical University, Kunming, 650500 Yunnan China; 2 Department of Orthopedic Surgery, The First People’s Hospital of Yunnan Province, The Affiliated Hospital of Kunming University of Science and Technology, The Key Laboratory of Digital Orthopaedics of Yunnan Provincial, Yunnan Lvweijia Expert Workstation, Kunming, 650034 China; 3 Department of Trauma Surgery, The Central Hospital of Wuhan, Tongji Medical College, Huazhong University of Science and Technology, Wuhan, 430000 Hubei China; 4 Department of Orthopedic Surgery, 920th Hospital of Joint Logistics Support Force of PLA, Kunming, 650032 Yunnan China

**Keywords:** Soft tissue defect, Rotation flap, Perforating branch of the proper artery, Distal finger pulp, Fingertip

## Abstract

**Background:**

Soft tissue defects in the hand may result from trauma, oncological procedures, or severe infections. This study aimed to introduce an innovative method for repairing soft tissue defects on the palmar side of the distal segment of the affected finger or fingertip. We explored this surgical method and its curative effect on the volar rotation pedicled flap base on a perforator of the palmar digital artery (VRPF-PPDA) for repairing ventral or fingertip soft tissue defects of the distal segment of the affected finger without impairing its main blood vessels.

**Methods:**

Between June 2019 and January 2021, 13 patients with finger pulp or fingertip soft tissue defects were treated with VRPF-PPDA. Flap survival rate, complication rate, two-point discrimination (2PD), and patient satisfaction were used to evaluate the efficacy of this method. The function of the affected finger was evaluated using the upper limb function evaluation method issued by the Trial Standards for Evaluation of Partial Function of the Upper Extremity of the Chinese Society for Surgery of the Hand of the Chinese Medical Association (CMA) and the Disabilities of the Arm, Shoulder, and Head (DASH) score, 6–12 months after the flap-based operation.

**Results:**

Thirteen patients (18 fingers) achieved complete flap survival. The finger pulp flap was full, and no complications occurred. 2PD checks of the flaps revealed that all of them were 4–10 mm in length. According to the Trial Standards for Evaluation of Partial Function of the Upper Extremity of the Chinese Society for Surgery of the Hand of the CMA, hand function was excellent in 12 patients (17 fingers) and good in one patient, with a mean DASH score of 26.05 ± 0.45. Eleven patients selected “excellent” on the subjective satisfaction survey, while the other two selected “good.”

**Conclusion:**

VRPF-PPDA surgery is a simple, effective, minimally invasive, and reliable method for repairing soft tissue defects in the distal finger pulp or fingertips. Optimal esthetic reconstruction and anatomical and functional repair can be achieved in fingers repaired using the VRPF-PPDA surgical approach.

## Background

Defects in the distal finger pulp and fingertip are common emergencies during hand surgery, and these soft tissue hand defects may result from trauma, oncological procedures, or severe infections [1]. Plastic and hand surgeons have always been faced with the challenges of achieving robust repair of the distal end of fingers, good soft tissue coverage to protect underlying structure, functional recovery as quickly and completely as possible, and obtaining the best esthetic effect [2]. A perforating flap was proposed by the Japanese researchers Koshima and Soeda in 1989 [3]. It is a flap comprising the perforator vessels as the direct source of blood supply. The perforating flap carries vascular perforators into the flap, and this differs from use of the random skin resection of the deep fascia as a source of blood supply or a necessary part of reflux. This flap is based on the blood supply to small perforator vessels, signifying a new development in microsurgical flap transplantation.

The stratum corneum of the palmar skin of the finger is thick, with a fat pad under the skin. Bone and skin were connected via vertical ligaments structures. The skin of the finger pulp contains threads that can increase its friction; thus, the functions of grasping, holding, and pinching are more stable, and the finger pulp is sensitive to these movements. These special attributes of the soft tissue of the distal finger pulp necessitate the use of the full thickness of the soft tissue to repair this extremity [4, 5]. Currently, several repair methods are available, including cross-finger [6], Thenar flaps [7], the first toe fibular free flap [8], partial second-toe transfers [9], and the dorsal flap of the first web [10]. However, there is no surgical method that considers the characteristics of minimally invasive, convenient, economical and anatomical repair involving physical similarity of tissues.

The soft tissue adjacent to the wound may provide the optimal solution for pedicle transfer of the flap donor area. In 2016, Zhou et al. employed the digital artery rotation flap to repair a fingertip skin defect connected to the digital artery pedicle, following soft tissue resection of the middle phalanx [11]. Fingertip rotation flap surgery is gradually becoming more widespread. Although the skin texture of the finger pulp repaired by this surgical method is similar to that of physiologically normal tissue, the blood supply of the flap is provided by the proper artery of the finger. Since the proper artery and nerve of the finger need to be dissected to enter the flap, the resulting surgical trauma is extensive. To overcome these surgical hurdles, a minimally invasive procedure was developed to repair the defect. This procedure involves cutting the second pulp of the finger; this allows avoidance of the main trunk of the proper digital artery (PDA), and selection of the perforator of the PDA as blood supply for the flap. This study aimed to analyze the anatomical feasibility of using the perforating branch of the proper artery as the pivot point to rotate the flap to the distal finger pulp and fingertip. We present our novel surgical technique and assess its outcome in a series of patients.

## Methods

### Patients

Between June 2019 and January 2021, soft tissue defects of the finger pulp or fingertip in 13 patients were repaired using a volar rotation flap pedicled with the perforator of the proper palmar digital artery (VRPF-PPDA), including ten males and three females, with an average age of 34.23 ± 12.94 years. Among them was one case of a three-finger pulp soft tissue defect, three cases of a two-finger pulp soft tissue defect, and the rest were single-finger pulp soft tissue defects. Each patient had bone exposure; five had nail bed injuries, and seven had distal phalanx fractures or defects. The proximal end of the defect was no longer than the distal fingertip, and neither side exceeded the midline of the finger (Table 1). All injured patients underwent preoperative routine blood examination, oblique hand X-ray examination, and emergency surgery and > 6 months of follow-up by the same group of doctors.

**Table 1:** Demographic date

Patient no.	Sex	Age (Y)	Injury finger	Defect size (cm^2^)	Mechanism of injury
1	M	27	LMF	1.7 cm * 2.1 cm	Crush
2	M	13	RMF	1.5 cm * 2.3 cm	Crush
3	M	48	RLF	1.5 cm * 1.9 cm	Crush
4	F	54	RIF	2.2.cm * 1.8 cm	Crush
5	M	49	LMF, LRF	2.1 cm * 1.9 cm/2.2 cm * 1.7.cm	Mechanical extrusion
6	M	37	RRF	2.1 cm * 1.8 cm	Door extrusion
7	M	47	LIF, LMF, LRF	1.9 cm * 1.3 cm/2.2.cm * 1.6 cm/2.0 cm * 1.8 cm	Mechanical extrusion
8	F	21	LRF	2.0 cm * 1.8 cm	Cut
9	M	25	LMF, LRF	2.3 cm * 1.9 cm/1.9 cm * 1.9 cm	Crush
10	M	26	RIF	2.1 cm*1.6 cm	Cut
11	M	40	RIF, RRF	1.6 cm * 1.7 cm/2.2 cm * 1.7 cm	Cut
12	F	17	LMF	2.4 cm * 1.8 cm	Crush
13	M	41	RMF	2.1 cm * 2.4 cm	Crush
Mean ± SD		34.23 ± 12.94		3.67 ± 0.57	

*LIF* Left index finger, *LMF* Left middle finger, *LRF* Left ring finger, *LLF* Left little finger, *RIF* Right index finger, *RMF* Right middle finger, *RRF* Right ring finger, *RLF* Right little finger

This clinical study was conducted with the approval of our institutional review board. We retrospectively analyzed patients with soft tissue defects of the fingertip or finger pulp. All patients were treated using the VRPF-PPDA.

### Anatomical basis of flap design

The common digital artery bifurcates into two palmar proper digital arteries at the level of the metacarpal head. The palmar PDA has several palmar and dorsal branches in each phalanx, and these branches converge into the palmar arch of the proximal phalanx, the palmar arch of the middle phalanx, and the arterial arch of the distal phalanx. The dorsal branch of the PDA gives off the joint branch, the medullary branch, and the dorsal arch of the distal finger. The PDA has two relatively large branches at the distal interphalangeal joint (DIP) level: one is located on the proximal side and is the main source of the transverse arch.

The perforating branch of the PDA is located far away from the transverse arch. Approximately 2–3 mm after the PDA separates, it is divided into a bifurcated “Y” shape and enters the skin to nourish the skin and soft tissue of the middle and distal parts of the finger pulp [12]. The number of perforators on the dominant side is greater than that on the non-dominant side. The ventral sensation of the second pulp of the finger and the proximal end of the distal phalanx mainly originates from the proper digital nerve (PDN) and its joint branch. The PDN runs toward the distal end, and the nerve endings retract from the soft tissue of the fingertip. The anatomy of the blood vessels and nerves forms the basis for flap survival and sensory recovery. Therefore, it is feasible to remove the ventral rotation flap without separating the PDA and only bring the DIP perforator of the PDA into the flap pedicle.

### Surgical treatment process

Wound debridement was performed using a tourniquet under the brachial plexus or by finger root nerve blocking anesthesia. The contaminated wounds were cleaned and disinfected. Approximately 1 mm of skin around the wound edge was removed using a surgical blade. From the outside to the inside, dirt and inactivated tissue were debrided from shallow to deep to create a clean wound, and the skin, nail bed, and distal fracture ends were trimmed.

*Flap design* The flap was designed on the palmar side of the middle segment of the injured finger according to the size and shape of the defect. The flap was pedicled with a surface projection of the perforators at the PDA joint of the proximal normal skin on the side of the wounded finger. The rotation point of the flap is often selected on the dominant side of the blood vessels. A rectangular flap is placed on the palmar side of the middle segment. The transverse width of the flap was approximately 3 mm longer than the longitudinal length of the wound, and its longitudinal length was approximately 2 mm longer than the width of the wound. The opposing side of the vascular pedicle was designed based on flap shape. The shapes of the distal defect edges of the wounds were similar. The distal side of the flap can also be cut as needed to the midline of the finger side, but should not exceed the midline.

**Fig. 1:**
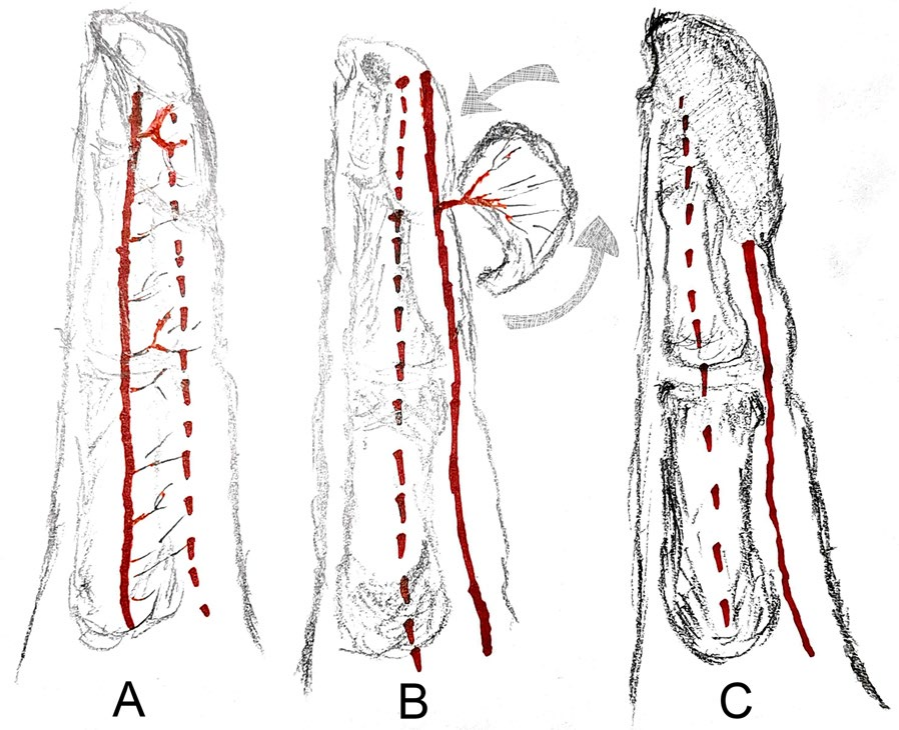
Schematic showing flap resection details. **A** Finger pulp soft tissue defect and distal phalanx exposed; **B** Flap carrying the perforator of the proper digital artery joint; **C** The flap was rotated 90 degrees after it was removed and sutured to the finger pulp wound

*Cutting of the flap* First, skin was cut from the side of the pedicle and the subcutaneous tissue; the superficial fascial layer was cut in depth; the flap was cut from the superficial layer of the pedicle side blood vessels and nerves, retaining the PDA and PDN. A small amount of tissue was covered, and the contralateral side was removed from the upper layer of the blood vessels and nerves. The flap was precisely resected in the superficial fascial layer without separating the main blood vessels and nerves. The flap was freed up to the distal end, and healthy skin and soft tissue of approximately 4–6 mm around the pedicle of the DIP were retained to ensure that the PDA perforator and PDN branch entered the flap. After appropriate dissociation to obtain sufficient rotation range, the flap was rotated 90° with the perforator at the joint of the PDA as the pedicle and sutured to the wound edge (Fig. 1).

*Flap donor site treatment* Skin grafts at the flap donor site can be divided into two categories: 1. The remaining tissue block of the severed finger is trimmed to full thickness. 2. When a tissue block is missing or unavailable, skin of the same size is removed from the medial upper arm and trimmed into full-thickness skin for skin grafting.

### Postoperative treatment

After the operation, the patient lay in bed for approximately 3 days with the hand fixed in the functional position and cushioned at the heart level. The dressing was windowed, and part of the flap was exposed to evaluate blood supply and to avoid the flap being pressed after bleeding. The dressing was replaced in time. Appropriate anticoagulation therapy and routine use of antibiotics was continued up to 5 days after the start of flexion and extension functional exercises. After approximately 2 weeks, the wound suture was removed and functional exercises were intensified to achieve timely tactile stimulation of the injured fingertips, until the patient entered recovery work [13].

### Postoperative functional evaluation

Postoperative flap survival rate, incidence of complications, flap fullness, 2PD, and a subjective patient satisfaction survey [9] were used to evaluate the flap. In a later follow-up, we added the self-administered Cold Intolerance Severity Score (CISS) to evaluate the cold intolerance of the injured and donor fingers [14]. Pain was evaluated using the following ratings: grade 1, no pain; grade 2, mild pain, not interfering with daily activities; grade 3, moderate pain allowing the patient to work normally, but with certain restrictions on the use of the hands due to pain; and grade 4, intense pain, with the patient unable to work or use hands. For the flap donor site, we increased the Tinel sign classification to grade 1, no signs of nerve stimulation; grade 2, mild tingling stimulation; grade 3, moderate stimulation, very uncomfortable; and grade 4, severe stimulation with the patient being unable to use the hand in response to any neuronal stimulation [15, 16]. The function of the affected limb was evaluated using the Disabilities of the Arm, Shoulder, and Head (DASH) score and upper limb function evaluation criteria issued by the Hand Surgery Society of the Chinese Medical Association (CMA) [17] from 6 to 12 months after the flap operation. All tests were performed by an independent senior hand surgeon.

## Results

The intraoperative blood loss was 5–8 mL, and operation time was confined to < 40 min. All flaps healed 12–17 days after the operation. No necrosis was observed in the 18 fingers of the 13 patients. One patient had local infection and exudation on the third postoperative day, and the wound healed after a change in dressing.

Eleven patients selected “excellent” on the subjective satisfaction survey, while the remaining two selected “good.” No patient required secondary surgery to reduce the flap volume. DIP flexion and extension activities were normal in all the patients. The flap survival rate was 100%. After half a year of follow-up, no obvious scar contracture was observed in the donor or recipient areas of the injured finger flap. A total of 92.3% of the patients said there was no difference in the shape and function of their affected fingers compared to the contralateral healthy fingers. Some patients even claimed that they had forgotten that the finger pulp was reconstructed via flap transplantation. All finger pulp flaps of the patients regained sensation, 2PD was 4–10 mm, and 13 patients had a mild CISS score (0–25 points). The pain scores of all patients were grade 1, and there was no interference with their daily work or lives. Among the 13 patients with Tinel's sign at the donor site, 18 fingers were graded, of which 16 were grade 1, and two were grade 2. After 6–12 months of follow-up, according to the upper limb function evaluation criteria issued by the Hand Surgery Society of the CMA [17], 12 patients (17 fingers) reported excellent hand function, and one patient showed good hand function, with a mean DASH [18] score of 26.05 ± 0.45 (Table 2).

**Table 2:** Postoperative findings and final follow-up

Patient no	2PD (mm)	DASH	Function evaluation by Hand Surgery Society of the Chinese Medical Association	CISS	Pain scores	Subjective satisfaction	Tinel sign classification to grade in the donor site
1	6	25.86	Excellent	Mild	No pain	Excellent	Grade 1
2	7	25.86	Excellent	Mild	No pain	Excellent	Grade 1
3	7	25.86	Excellent	Mild	No pain	Excellent	Grade 1
4	8	25.86	Good	Mild	No pain	Excellent	Grade 1
5	5/7	25.86	Excellent	Mild	No pain	Excellent	Grade 2 (LMF)
6	7	25.86	Excellent	Mild	No pain	Excellent	Grade 1
7	6/7/10	27.58	Excellent	Mild	No pain	Good	Grade 2 (LRF)
8	5	25.86	Excellent	Mild	No pain	Excellent	Grade 1
9	5/8	25.86	Excellent	Mild	No pain	Excellent	Grade 1
10	4	25.86	Excellent	Mild	No pain	Excellent	Grade 1
11	7/8	25.86	Excellent	Mild	No pain	Good	Grade 1
12	5	25.86	Excellent	Mild	No pain	Excellent	Grade 1
13	7	26.72	Excellent	Mild	No pain	Excellent	Grade 1

*LMF* Left middle finger; *LRF* Left ring finger, *2PD* Statically measured two-point discrimination, *DASH* The Disabilities of the Arm, Shoulder, and Head score, *CISS* Cold Intolerance Severity Score

### Case 1

**Fig. 2:**
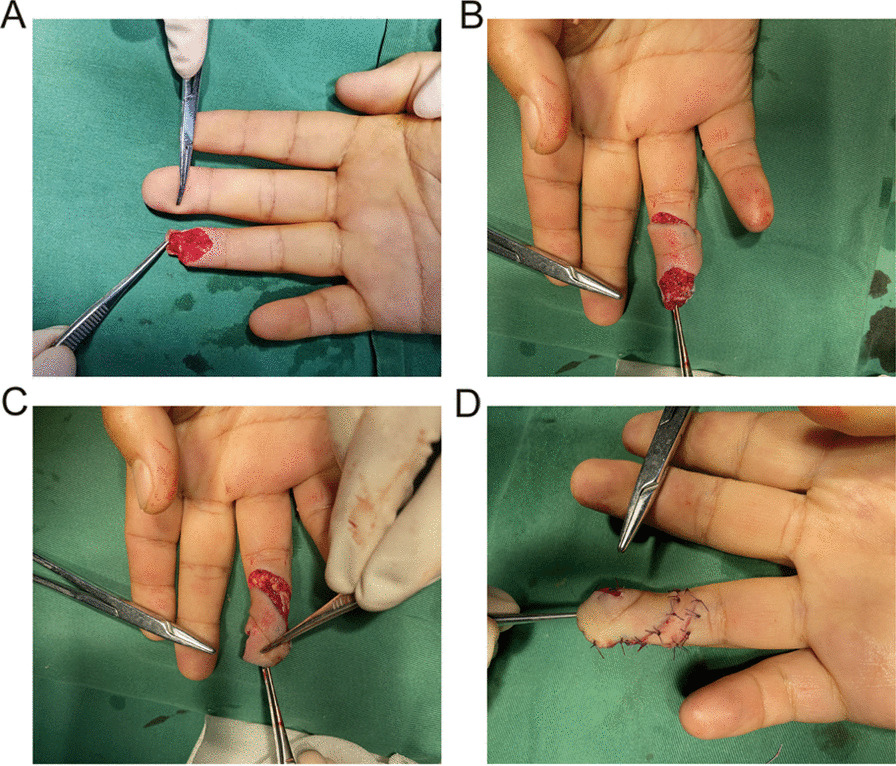
Details of the injury and surgical process. **A** The right finger was clipped by a car door, and the soft tissue mass of the distal segment was damaged. There was no replantation; **B** The right ring finger palmar rotary flap pedicled with the perforator of the proper palmar artery of the finger was used to repair the soft tissue defect. The edge of the flap donor area was cut, and the dominant pedicle of the blood vessel was preserved; **C** The ring finger flap was cut, and the pedicle of approximately 5 mm length on the radial side was retained so that the cutaneous branch of the proper digital artery could enter the flap to provide blood supply. The flap was rotated to the distal end to cover the distal wound of the finger pulp; **D** The flap was sutured to the recipient area, and free skin grafting was performed on the donor area of the flap

**Fig. 3:**
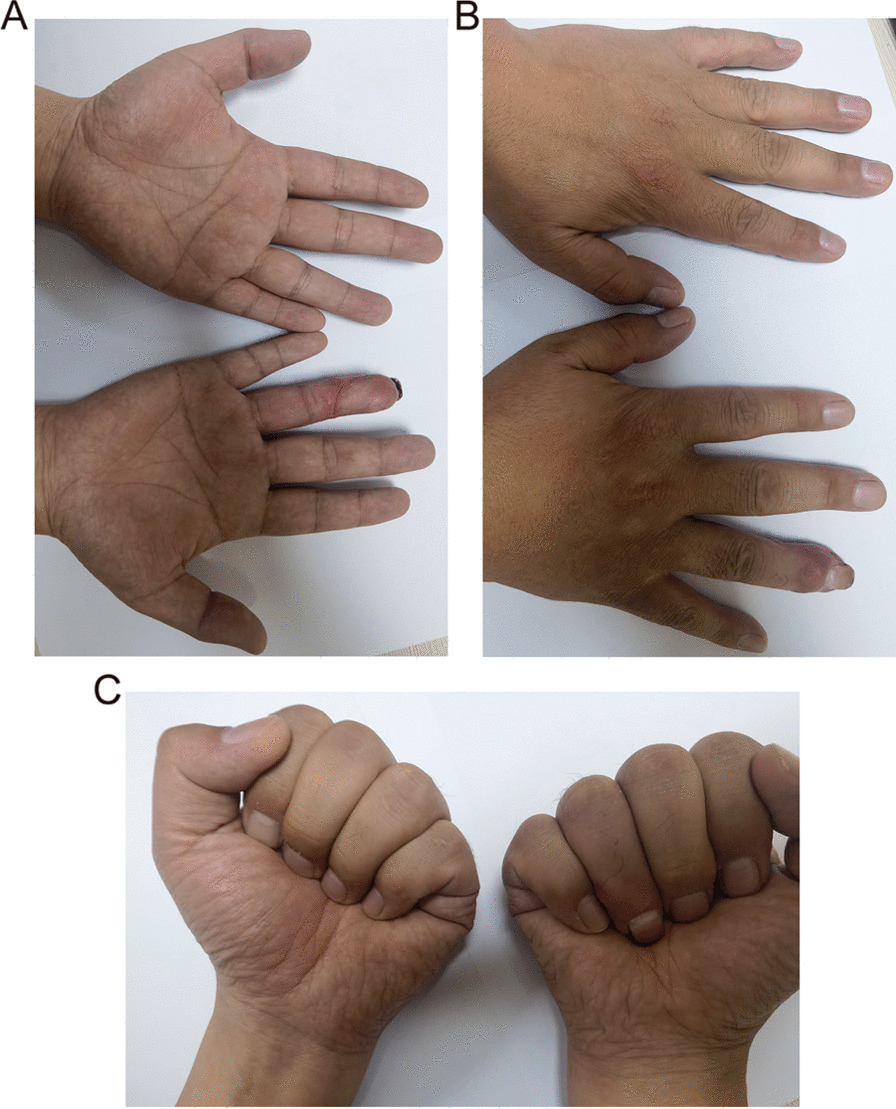
Appearance and function of the injured finger flap were reviewed 9 weeks after surgery. **A** After repair of the injured finger with the flap, the finger pulp was full, and the appearance was satisfactory; **B** No difference in finger extension function was observed between the injured side and the healthy side; **C** The hand grip of the injured side was normal, and ring finger flexion activity was the same as that of the healthy side's finger body

**Fig. 4:**
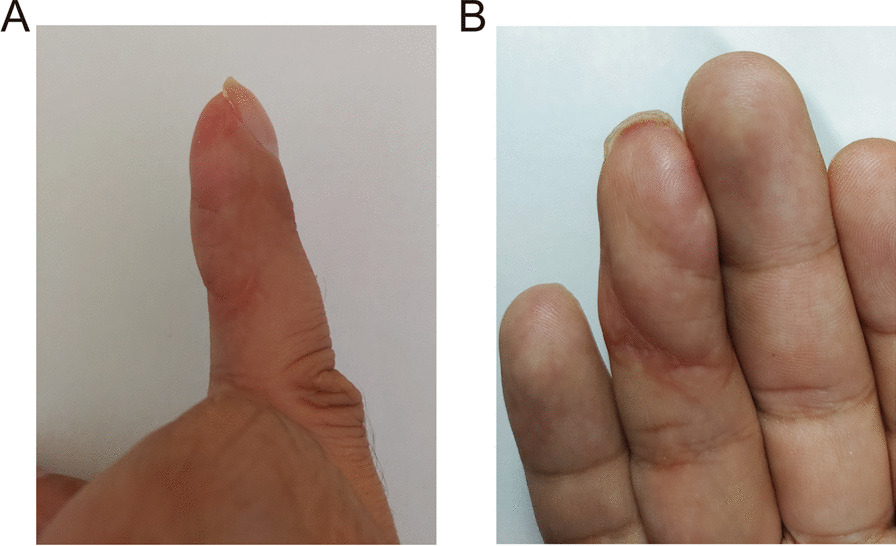
The appearance of the finger 6 months after surgery. **A** From the radial side of the finger, the finger pulp was full; **B** The color and texture of the finger pulp after surgery were nearly normal

**Fig. 5:**
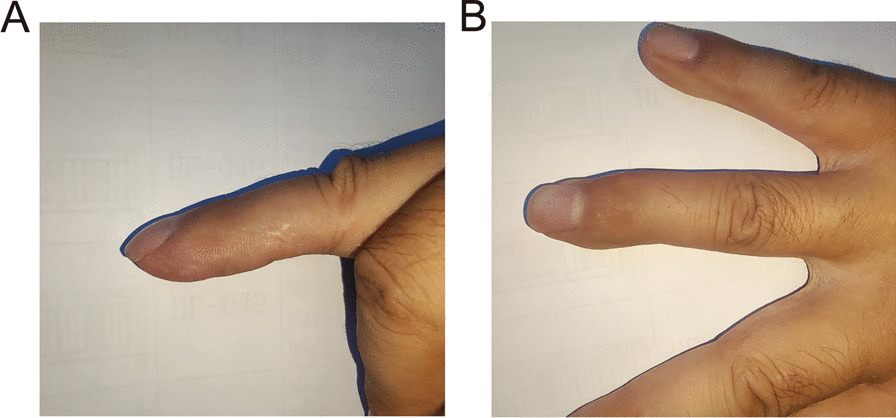
Appearance of the finger 1 year after the operation. **A** From the side of the finger, the finger pulp that underwent surgery was full, and without scar hyperplasia; **B** After the operation, the color and texture were nearly normal

A 37-year-old man was injured on the right ring finger by a car door. The exposed distal phalanx was observed in the wound, and a palmar rotation flap pedicled with a perforating branch of the proper palmar digital artery was used to repair the defect. The flap size was 2.1 cm by 1.8 cm (Fig. 2). The flap survived completely. Clinical evaluation of 9 weeks of postoperative follow-up showed that the flap scar was not obvious, there was no scar contracture, finger pulp of the patient was full, flexion and extension function of the DIP was normal, and the ring finger was unaffected when holding objects (Fig. 3). The static and moving 2PDs of the flap were 7.0 and 5.0 mm, respectively. The CISS score was mild at 6 months after the operation. The pain scores of the injured fingers were all grade 1, and the Tinel’s sign of the donor area was grade 1 (Fig. 4). According to the upper limb function evaluation criteria issued by the Hand Surgery Society of the CMA, hand function was excellent, and the DASH score was 25.86 (Fig. 5).

### Case 2

**Fig. 6:**
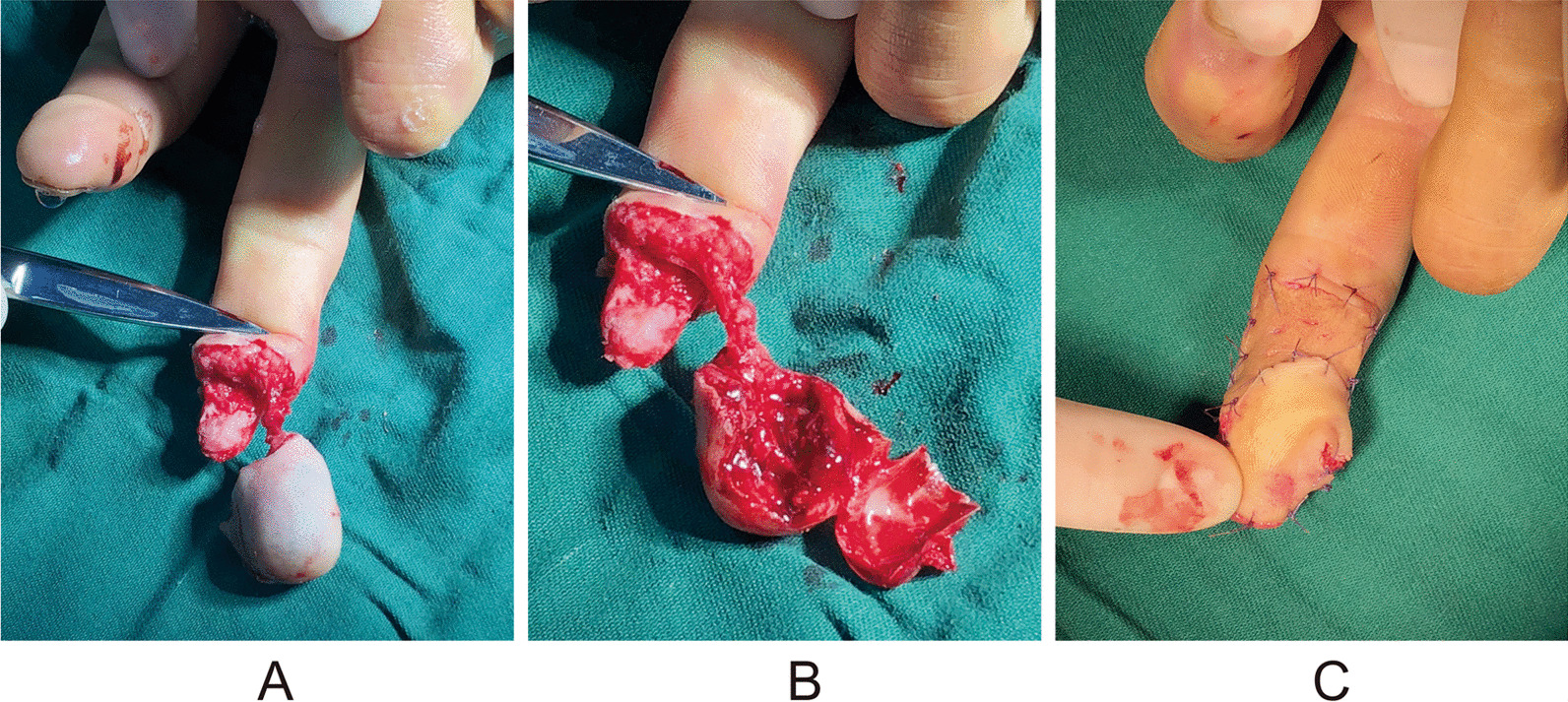
Details of injury and surgical process for case number 2. **A** The soft tissue mass of the distal phalanx of the middle finger was separated from the finger body, and only the proper digital artery of one side of the contusion was retained. The separated tissue mass had subcutaneous congestion, soft tissue contusion, and no blood supply; **B** Distal phalanx exposed to show the lack of soft tissue coverage; **C** The volar rotation flap pedicled with the perforator of the proper palmar digital artery was used to repair the defect of the distal phalanx. After the repair, the finger pulp was full, and free skin grafting was performed on the flap donor site

A 41-year-old right-handed male patient had the middle finger of his right hand clipped off by a machine from the middle of the distal segment onward, and there was no replantation far from the severed finger. Bone exposure was also observed in the wound. No distal phalanx fractures were observed. The nail fell off, exposing the nail bed. A palmar rotation flap pedicled with the perforating branch of the proper palmar artery of the finger was used to repair the defect. The length of the scar was extensive and completely preserved, and the integrity of the reconstructed fingertip and pulp was as full as that of the normal finger. After 3 months of follow-up, the static and moving 2PDs of the flap were 7.0 and 6.0 cm, respectively. There was no significant difference in the flexion and extension functions between the affected and the healthy sides, and the CISS score was mild. The pain scores of the injured fingers were all grade 1, and Tinel’s sign at the donor site was grade 1. According to the upper limb function evaluation criteria issued by the Hand Surgery Society of the CMA, hand function was excellent, with a DASH score of 26.72 (Fig. 6).

## Discussion

Conventional repair methods for finger pulp soft tissue defects include the V–Y flap, cross-finger flap, proximal finger flap [19], digital artery inverted flap, dorsal flap of the first web [10], partial second toe transfer [9], and covering the wound with biomaterials to repair it [20]. Various flaps require different adaptation conditions [21]. The V–Y advancement flap is no longer indicated when the distal volar defect area of the finger exceeds 7 mm in length [2]. In the V–Y advancement flap [22], the wound is located at the center of the finger pulp. After wound healing, the Y-shaped scar of the finger pulp considerably impacts the appearance of the healed area. The VRPF-PPDA procedure completely neutralizes the impact of the wound located at the center of the finger pulp on the appearance of the healed area [2]. The cross-finger flap was originally proposed as a random-pattern flap [19]. Thus, the flap is safe and reliable. The skin can be removed to cover the entire defect of the distal finger pulp. The resection area is limited to the skin on the dorsal side of the donor finger. However, the cross-finger flap procedure must be completed in two operations: flap transplantation and an additional pedicle-cutting operation. When the donor finger flap is cut, it is necessary to plant the skin on the tendon aponeurosis, as there is less soft tissue in the fascial layer. The central area of the skin graft may increase the possibility of skin necrosis owing to blood accumulation and the presence of uneven stress [23, 24]. Using a proximal finger flap [20], a digital artery inverted flap or a dorsal flap of the first web [10] to repair the defect of the distal finger pulp and fingertip is tantamount to demolishing an expensive wall and constructing a cheaper wall to replace it; the gain is lost. This approach does not conform to the principles of flap economics. The free flap procedure includes a partial second toe transfer [9, 25] and the inclusion of a first toe fibular free flap. It is widely used; however, microsurgical vascular anastomosis technology limits any further increase in its application. Recently, biological materials have been used for wound repair. Owing to the minimal trauma involved in their use and few complications, robust repair results have been achieved. However, this method increases the burden on patients, and the treatment period is longer than the operation itself, and this limits their application [21, 28].

The finger pulp rotary flap [11] is a pedicled flap nourished by the palmar proper artery of one finger and is widely used to repair finger pulp soft tissue defects in the distal segment of the hand. However, the flap needs to sacrifice or free one side of the PDA and the PDN. The fingertip rotation flap must completely free the flap [11], and only the vascular pedicle of the finger artery should be retained. After the flap is rotated by 90°, the wound in the flap donor area is exposed on the finger side, and free skin grafting is required on this side [26]. In our surgical approach, the donor-site skin defect on the palmar side of the finger is more conducive to the application of a full-thickness skin graft. After the flap is removed, more soft tissue remains in the donor area than in the fingertip rotation flap [27]: this is more conducive to the survival of the skin graft and does not cause a significant depression after the skin graft heals.

Generally, for a wound located far from the transverse striation of the DIP, the length of the wound should not be greater than the length of the line connecting the two sides of the middle finger pulp, and this is the best indication for VRPF-PPDA. High-tension sutures increase flap tension, the venous return pressure of the flap, and the incidence of hook nail deformity. Soft tissue wounds should be evaluated after debridement to maintain the fullness of the distal finger pulp and reduce flap tension. The flap design should be more prominent than that of the wound defect. Thorough debridement is crucial to preventing infection and is also important for flap removal. Attention should be paid to protecting the PDA and PDN on the rotating side. Some soft tissue should be left on the surface of the nerve and blood vessels to prevent the skin graft from surviving or forming scars that compress the blood vessels and nerves, leading to nerve sensitivity. VRPF-PPDA is a relatively simple procedure. The operation can be completed without a microscope or a surgical magnifying glass because of minimal blood vessel disturbance [28]. The skin is highly similar to the normal finger pulp skin. There is no additional wounding of the surgical area, and the trauma is minimal. Postoperative flap reflux is good; extra-arterial pressurization and venous super-reflux are not required. The branch of the proper nerve joint of the finger enters the flap, and the sensory function of the flap improves after surgery. There is no significant difference in the 2PD examination of the flap carrying the PDN trunk. However, the application of VRPF-PPDA for repairing soft tissue defects of the distal thumb requires further investigation.

## Conclusion

The VRPF-PPDA procedure, that involves only a perforating branch of the PDA, is a simple, effective, minimally invasive, and reliable method for repairing soft tissue defects in the distal finger pulp or fingertip. Optimal aesthetic reconstruction with anatomical and functional repair can be achieved using the VRPF-PPDA surgical approach to repair the distal finger pulp or fingertip.

## Data Availability

The datasets used and/or analyzed during the current study are available from the corresponding author on reasonable request.

## Abbreviations

VRPF-PPDA: Volar rotation pedicled flap base on a perforator of the palmar digital artery
2PD: Two-point discrimination
DASH: Disabilities of the arm, shoulder, and head
DIP: Distal interphalangeal joint
PDA: Proper digital artery
PDN: Proper digital nerve
CISS: Cold intolerance severity score

## References

[1] Battiston B, Fulchignoni C (2023). Soft tissue defects of the hand: etiology and classification. Plast Aesthet Res.

[2] Qin JZ, Wang PJ (2012). Fingertip reconstruction with a flap based on the dorsal branch of the digital artery at the middle phalanx: a simple and reliable flap. Ann Plast Surg.

[3] Narushima M, Yamasoba T, Iida T, Yamamoto T, Yoshimatsu H, Hara H, Oshima A, Todokoro T, Kikuchi K, Araki J (2011). Pure skin perforator flap for microtia and congenital aural atresia using supermicrosurgical techniques. J Plast Reconstr Aesthet Surg.

[4] Wang X, Lv J, Liu S, Xu S, Yan G (2022). Effect of dorsal nerve fascial island flap on repairing distal soft tissue defects at the proximal segment of the index, middle, ring, and little fingers. J Orthop Surg Res.

[5] Wang P, Zhou Z, Dong Q, Jiang B, Zhao J (2011). Reverse second and third dorsal metacarpal artery fasciocutaneous flaps for repair of distal- and middle-segment finger soft tissue defects. J Reconstr Microsurg.

[6] Cam N, Kanar M (2022). Are cross finger and thenar flaps effective in the treatment of distal finger amputations with the reposition-flap method?. Jt Dis Relat Surg.

[7] Polatsch DB, Rabinovich RV, Beldner S (2017). The double thenar flap: a technique to reconstruct 2 fingertip amputations simultaneously. J Hand Surg Am.

[8] Zhang J, Xie Z, Lei Y, Song J, Guo Q, Xiao J (2008). Free second toe one-stage-plasty and transfer for thumb or finger reconstruction. Microsurgery.

[9] Brunelli F, Spalvieri C, Rocchi L, Pivato G, Pajardi G (2008). Reconstruction of the distal finger with partial second toe transfers by means of an exteriorised pedicle. J Hand Surg Eur.

[10] Pagliei A, Rocchi L, Tulli A (2003). The dorsal flap of the first web. J Hand Surg Br.

[11] Zhou JS, Yang P, Song KF, Li QS, Wei X, Hu XF, Li J (2016). Digital artery rotation flap in the repair of fingertip skin defect. Journal of Traumatic Surgery.

[12] Petchprapa CN, Vaswani D (2019). MRI of the fingers: an update. AJR Am J Roentgenol.

[13] Graham D, Sivakumar B, Piñal FD (2022). Triangular vascularized free fibula flap for massive carpal reconstruction. J Hand Surg Am.

[14] Carlsson I, Cederlund R, Höglund P, Lundborg G, Rosén B (2008). Hand injuries and cold sensitivity: reliability and validity of cold sensitivity questionnaires. Disabil Rehabil.

[15] Poole JL (2011). Measures of hand function: arthritis hand function test (AHFT), Australian Canadian osteoarthritis hand index (AUSCAN), Cochin hand function scale, functional index for hand osteoarthritis (FIHOA), grip ability test (GAT), Jebsen hand function test (JHFT), and Michigan hand outcomes questionnaire (MHQ). Arthritis Care Res.

[16] Duruöz MT, Poiraudeau S, Fermanian J, Menkes CJ, Amor B, Dougados M, Revel M (1996). Development and validation of a rheumatoid hand functional disability scale that assesses functional handicap. J Rheumatol.

[17] Pan DD, Gu YD, Shi D, Shou KS (2000). Trial standard of upper limb function evaluation by hand surgery society of Chinese Medical Association. Chin J Hand Surg.

[18] Belyea C, Pulos N, Ezaki M, Wall L, Mills J, Beckwith T, Oishi SN (2020). Effect of distal Ulna Osteochondroma excision and distal ulnar tether release on forearm deformity in preadolescent patients with multiple hereditary exostosis. J Pediatr Orthop.

[19] Rabarin F, Saint Cast Y, Jeudy J, Fouque PA, Cesari B, Bigorre N, Petit A, Raimbeau G (2016). Cross-finger flap for reconstruction of fingertip amputations: long-term results. Orthop Traumatol Surg Res.

[20] Fulchignoni C, Rocchi L, Cauteruccio M, Merendi G (2022). Matriderm dermal substitute in the treatment of post traumatic hand's fingertip tissue loss. J Cosmet Dermatol.

[21] Meyer-Marcotty MV, Kall S, Vogt PM (2007). Neurovascular flaps for the reconstruction of fingertip injuries. Unfallchirurg.

[22] Lim JX, Chung KC (2020). VY advancement, thenar flap, and cross-finger flaps. Hand Clin.

[23] He B, Liu J, Pang V, Zhu L, Huang Y, Wang Z, Xu Y, Zhu Z, Wang K (2019). Anatomical and clinical comparison of small free flaps for repairing finger skin defects. Ann Plast Surg.

[24] Xie H, Fang Q, Zhang D (2021). Flow-through flap with wrist epithelial branch of ulnar artery for repair of finger soft tissue defect: a case series. Am J Transl Res.

[25] Woo SH, Lee GJ, Kim KC, Ha SH, Kim JS (2006). Cosmetic reconstruction of distal finger absence with partial second toe transfer. J Plast Reconstr Aesthet Surg.

[26] Besmens IS, Guidi M, Frueh FS, Uyulmaz S, Lindenblatt N, Reissner L, Calcagni M (2020). Finger reconstruction with dorsal metacarpal artery perforator flaps and dorsal finger perforator flaps based on the dorsal branches of the palmar digital arteries: 40 consecutive cases. J Plast Surg Hand Surg.

[27] Chen C, Tang P, Zhang X (2013). Treatment of soft-tissue loss with nerve defect in the finger using the boomerang nerve flap. Plast Reconstr Surg.

[28] Zaidenberg EE, Farias-Cisneros E, Pastrana MJ, Zaidenberg CR (2017). Extended posterior interosseous artery flap: anatomical and clinical study. J Hand Surg Am.

